# 

**Published:** 1921-01-01

**Authors:** 


Ja 11 21 S

### THE HOSPITAL

**The Modern Newspaper of Administrative Medicine and Institutional Life.**

Telegraphic Address: OSPEDALE, RAND, LONDON. Telephone Number: 2734 GERRARD.

No. 1804, Vol. LXIX. | Saturday, January 1st, 1921. | PRICE TWOPENCE.

